# Cranial Neurolymphomatosis Presenting With Trigeminal Neuralgia and Facial and Vagus Nerve Palsies Visualized on Fluorodeoxyglucose Positron-Emission Tomography (FDG PET)

**DOI:** 10.7759/cureus.94240

**Published:** 2025-10-09

**Authors:** Pak Lai Tsoi, Michelle Ho Yan Cheung

**Affiliations:** 1 Nuclear Medicine, Pamela Youde Nethersole Eastern Hospital, Hong Kong, HKG

**Keywords:** bell's palsy, diffuse large-b-cell lymphoma, fluorodeoxyglucose positron-emission tomography, neurolymphomatosis, trigeminal neuralgia

## Abstract

Neurolymphomatosis (NL) is a rare and severe complication of lymphoma, characterized by lymphoma disease involvement in the central or peripheral nervous system. It is often associated with non-Hodgkin lymphoma, particularly diffuse large B-cell lymphoma (DLBCL), and is more frequently encountered in the setting of relapse. We report a case of a 68-year-old woman with stage IV DLBCL who relapsed after first-line treatment. She developed neurological manifestations, including trigeminal neuralgia, facial and vagus nerve palsies on relapse. ^18^F-fluorodeoxyglucose (FDG) positron emission tomography (PET) was performed. Florid FDG hypermetabolism was seen at branches of the left trigeminal nerve, left-sided pons, and left jugular foramen. Associated vocal cord palsy resultant from vagus nerve involvement was also visualized on FDG PET. Our case demonstrates the characteristic FDG PET imaging findings of cranial nerve NL, as well as highlights the importance of maintaining high clinical vigilance for NL and the usefulness of FDG PET imaging in the evaluation of NL.

## Introduction

Neurolymphomatosis (NL) is characterized by lymphoma disease involvement in the central or peripheral nervous system [1]. It is more frequently encountered in cases with disseminated or relapsed disease, rather than during initial presentation [2]. NL is mostly associated with non-Hodgkin lymphoma, particularly diffuse large B-cell lymphoma (DLBCL) [3]. Relapses involving the central nervous system are rare (around 2-5%), but carry devastating morbidity and prognosis [4]. Biopsy is the gold standard for diagnosis, but it is often technically challenging, with limited sensitivity and a significant risk of permanent nerve damage [5]. Imaging evaluation for suspected cases of NL mainly consists of ^18^F-fluorodeoxyglucose (FDG) positron emission tomography (PET) and MRI. Differential diagnoses for NL include herpes zoster, radiation neuritis/plexopathy, nerve root compression, paraneoplastic and lymphoma-associated vasculitis [6]. We report a case of neurolymphomatosis involving multiple cranial nerves, presenting with unilateral trigeminal neuralgia, Bell’s palsy, and vocal cord palsy, visualized on FDG PET.

## Case presentation

A 68-year-old woman presented with a left nasal mass. Biopsy of the mass showed diffuse large B-cell lymphoma (non-germinal center B-cell (GCB), non-double expressor subtype). Staging FDG PET showed stage IV disease involvement, including bilateral adrenals. She achieved complete metabolic response following a first-line chemotherapy regimen that consisted of rituximab, cyclophosphamide, hydroxydaunorubicin, vincristine, and prednisolone (R-CHOP), in accordance with international guidelines [7]. However, the disease recurred at a facial nodule (biopsy-proven) two months after achieving complete metabolic response. Aside from the facial nodule, she complained of concurrent neurological symptoms at the time of disease relapse, including left-sided facial pain, left facial muscle weakness, left-sided epiphora, and hoarseness of voice. Clinical evaluation showed touch allodynia affecting the mandibular (V3) division of the left trigeminal nerve (CN V), and pinprick hypoalgesia over the left ophthalmic (V1), maxillary (V2), and V3 divisions, suggestive of trigeminal neuralgia. Left masseter and frontalis weakness suggestive of left facial nerve palsy, as well as left-sided vocal cord paralysis, were also present. These neurological symptoms deteriorated despite the commencement of second-line chemotherapy consisted of methotrexate, cytarabine, thiotepa, and rituximab (MATRix) [8].

FDG PET was performed to evaluate the interim response to second-line treatment. The FDG PET was performed by a Discovery 690 scanner (GE Healthcare, Chicago, IL, USA), with acquisition protocols referencing international guidelines [9]. One bed of dedicated brain PET/CT images was acquired, performed immediately after the usual scan range (from skull base to thighs) at around 80 minutes.

**Figure 1:**
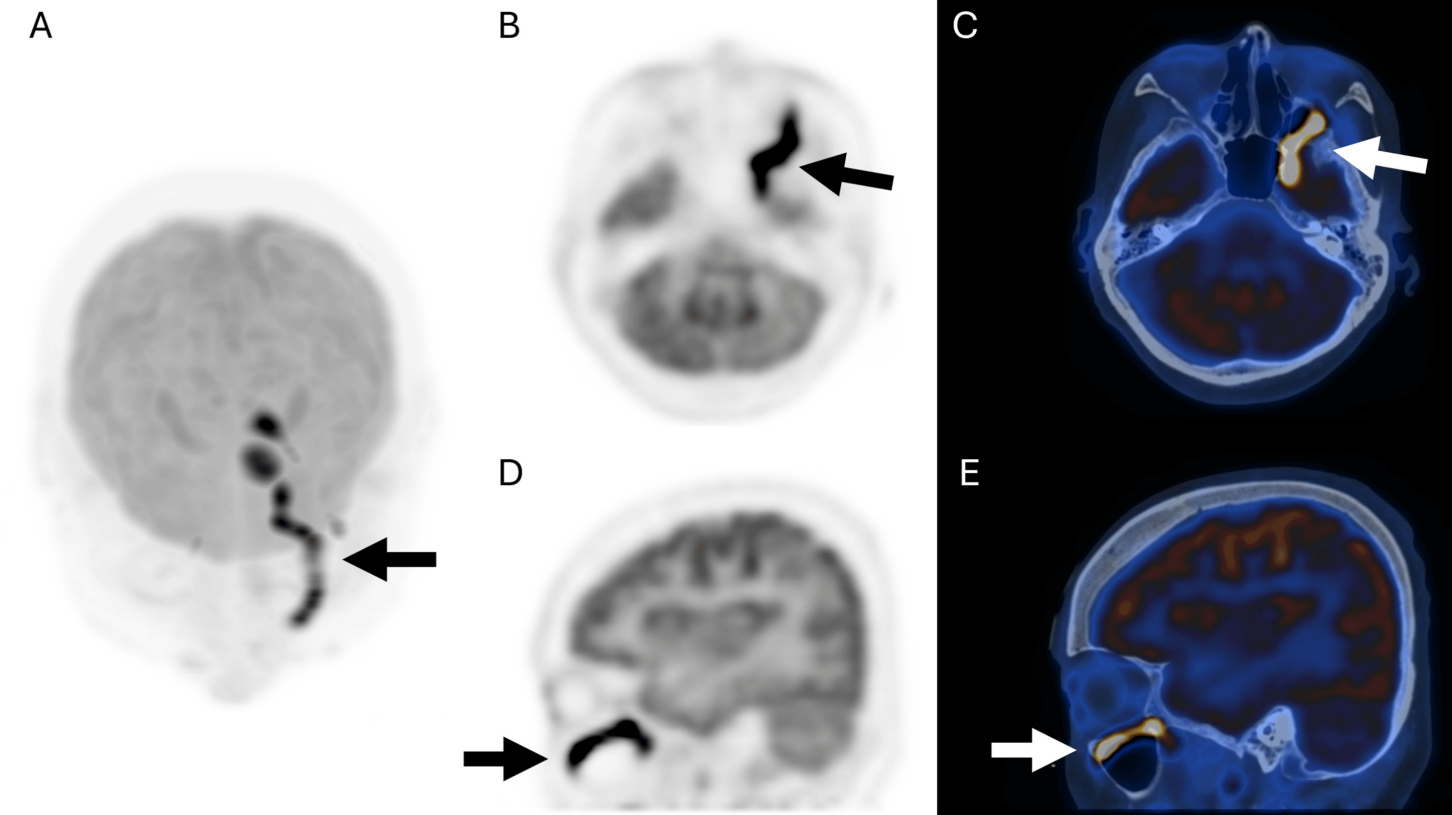
Brain maximum intensity projection image (Top-down view, A) shows multiple hypermetabolic lesions, most strikingly along the maxillary branch (V2) of the left trigeminal nerve. PET-only and PET/CT fusion images showing linear hypermetabolism at left V2 along the inferior orbital fissure (Axial: B-C; Sagittal: D-E).

**Figure 2:**
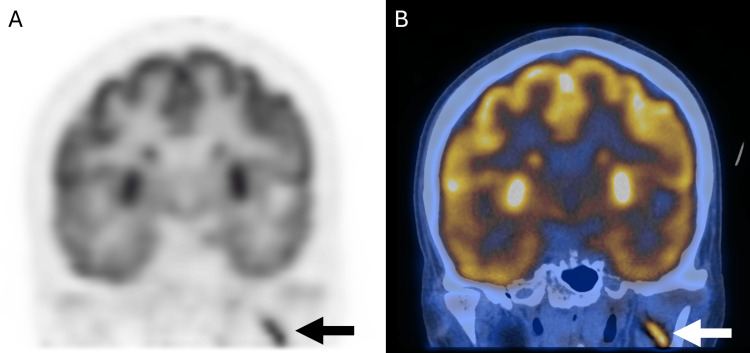
PET-only (A) and PET/CT fusion (B) coronal images showing linear hypermetabolism along the mandibular branch (V3) of the left trigeminal nerve

**Figure 3:**
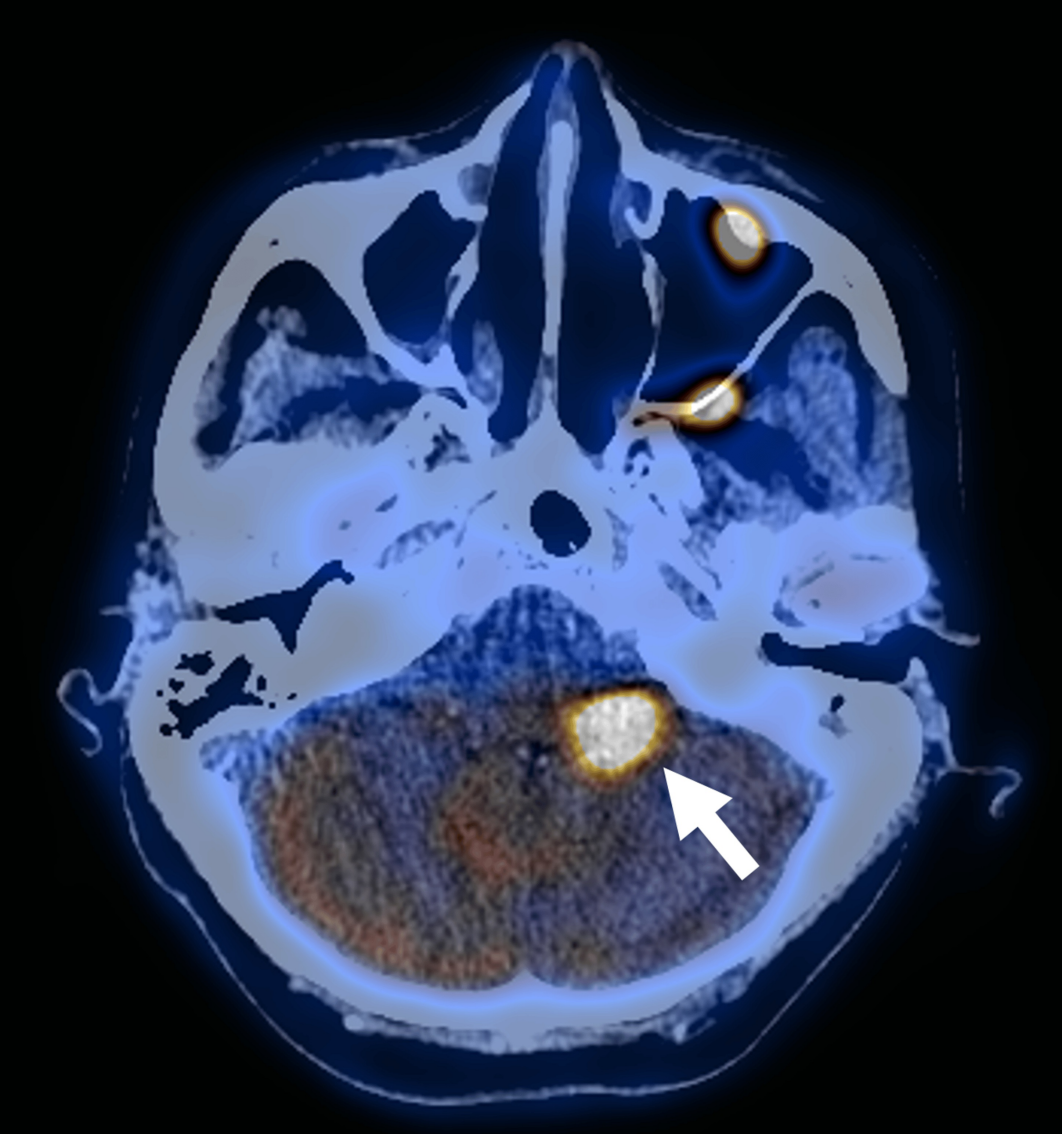
PET/CT fusion axial image showing focal hypermetabolism involving the left side of pons

**Figure 4:**
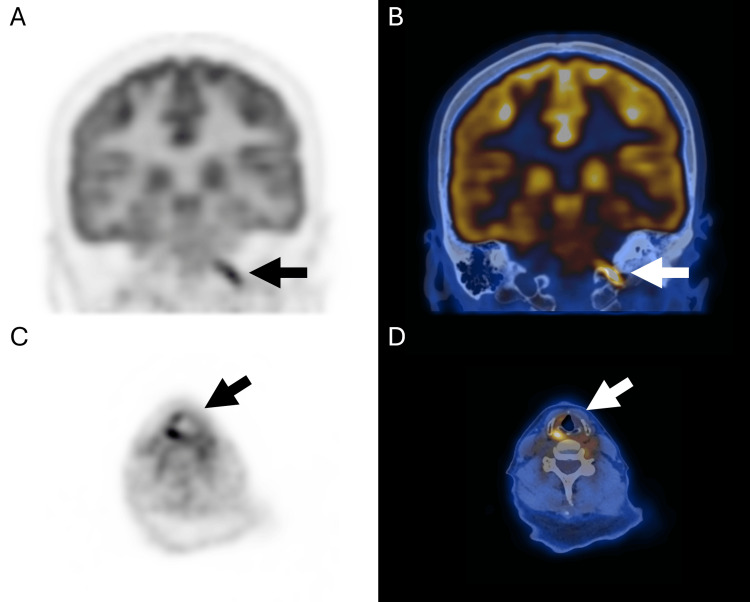
PET-only and PET/CT fusion coronal images showing linear hypermetabolism at the left jugular foramen (A-B). PET-only and PET/CT fusion axial images showing asymmetrical reduced FDG activity over the left vocal cord (C-D)

Segments of marked FDG hypermetabolism in linear configurations were seen along V2 (Figure 1) and V3 of CN V (Figure 2), accounting for left trigeminal neuralgia. Focal marked FDG hypermetabolism at the left-sided pons (Figure 3) likely accounts for left facial nerve palsy. Short linear hypermetabolism at the left jugular foramen, along with reduced FDG metabolism at the left vocal cord, together represent vagus nerve involvement with left vocal cord palsy (Figure 4).

Findings of intracranial disease progression from FDG PET prompted a change of treatment to nivolumab (immunotherapy) and whole brain radiotherapy (WBRT). Subsequent PET exam (images not included in this report) showed metabolic resolution of these intracranial lesions, indicating complete metabolic response.

## Discussion

NL can present in various clinical patterns, namely painful or painless polyneuropathy, cranial neuropathy, and peripheral mononeuropathy. Progression can be in a relapsing-remitting or progressive pattern. Around 20% of patients with NL present with isolated cranial neuropathy, which includes presentations such as “Bell’s palsy”, abducens, oculomotor, and trigeminal neuropathy, hearing loss, and vocal cord paralysis [1]. In our case, the patient presented with multiple cranial neuropathies (Bell palsy, trigeminal neuralgia, hearing loss, and vocal cord paralysis) in the setting of proven lymphoma recurrence elsewhere (at a facial nodule). The pattern of FDG hypermetabolism along the affected nerves is similar to reported cases by Canh et al. [10].

MRI and PET/CT are the main modalities for imaging evaluation of NL. MRI findings suggestive of NL include nerve enlargement/ thickening, T2-weighted hyperintensity, contrast enhancement, and restricted diffusion on diffusion-weighted sequences [11,12]. Characteristic imaging findings of NL involvement on FDG PET include linear/fusiform FDG uptake along nerves of variable avidity [13], often with no clear morphological abnormality on corresponding CT images [10]. According to the International Primary CNS Lymphoma Collaborative Group, FDG PET may be more sensitive than MRI in the diagnosis of neurolymphomatosis (84% vs 77%) [2]. FDG PET is also a validated tool in the response assessment of NL following systemic treatment [13].

Our case highlights the importance of maintaining high clinical vigilance for NL in patients with a lymphoma history, particularly when neurological deficits are encountered in the setting of relapsed/ recurrent disease. Timely FDG PET examination with inclusion of brain images is useful for the prompt diagnosis of NL. Limited whole-body imaging (i.e., base of skull to mid-thigh) is often the routine scanned range for oncological FDG PET [9]. Brain images should therefore be included to ensure coverage of all potential NL lesions when NL is clinically suspected. In addition to the detection of FDG-hypermetabolic lesions suggestive of NL, close attention should also be paid to screen for accessory findings suggestive of cranial nerve pathology associated with NL. These accessory findings visible on FDG PET usually consist of muscle atrophy and overcompensation (e.g., vocal cord palsy in our case), and are well illustrated in a pictorial essay by Raslan et al. [14].

## Conclusions

NL is a rare but debilitating manifestation of lymphoma, more often seen in the relapse/recurrent setting. The reported case highlighted the importance of maintaining a high clinical vigilance towards neurological symptoms in lymphoma patients and the value of FDG PET in the diagnosis of NL. The interesting images included in this case report demonstrated the characteristic FDG PET appearance of NL affecting multiple cranial nerves, which correlated with clinical findings of trigeminal neuralgia, Bell’s palsy, and vocal cord palsy. In addition to the identification of hypermetabolic lesions indicative of NL, efforts should also be made to screen for accessory findings suggestive of cranial nerve pathology associated with NL.
